# Astragaloside IV Enhances Melanogenesis via the AhR-Dependent AKT/GSK-3*β*/*β*-Catenin Pathway in Normal Human Epidermal Melanocytes

**DOI:** 10.1155/2020/8838656

**Published:** 2020-12-15

**Authors:** Baoyi Liu, Yongyi Xie, Zhouwei Wu

**Affiliations:** ^1^Department of Dermatology, Shanghai General Hospital, Shanghai Jiao Tong University School of Medicine, Shanghai 200080, China; ^2^Department of Dermatology, Shanghai General Hospital, Shanghai Jiao Tong University, Shanghai 200080, China

## Abstract

*Astragalus membranaceus* root has been widely used for repigmentation treatment in vitiligo, but its mechanism is poorly understood. We sought to investigate the effect of astragaloside IV (AS-IV), a main active extract of the *Astragalus membranaceus* root, on melanin synthesis in normal human epidermal melanocytes (NHEMs) and to elucidate its underlying mechanisms. Melanin content, tyrosinase activity, qPCR, western blot, and immunofluorescence were employed. Specific inhibitors and small interfering RNA were used to investigate the possible pathway. AS-IV stimulated melanin synthesis and upregulated the expression of melanogenesis-related genes in a concentration-dependent manner in NHEMs. AS-IV could activate the aryl hydrocarbon receptor (AhR), and AS-IV-induced melanogenesis was inhibited in si-AhR-transfected NHEMs. In addition, we showed that AS-IV enhanced the phosphorylation of AKT and GSK-3*β* and nuclear translocation of *β*-catenin. AS-IV-induced MITF expression upregulation and melanin synthesis were decreased in the presence of *β*-catenin inhibitor FH353. Furthermore, AhR antagonist CH223191 inhibited the activation of AKT/GSK-3*β*/*β*-catenin signaling, whereas the expression of CYP1A1 (marker of AhR activation) was not affected by the AKT inhibitor in AS-IV-exposed NHEMs. Our findings show that AS-IV induces melanogenesis through AhR-dependent AKT/GSK-3*β*/*β*-catenin pathway activation and could be beneficial in the therapy for depigmented skin disorders.

## 1. Background

Vitiligo is a kind of depigmentation disease with about 0.1–2% prevalence in the world [1], characterized by patchy depigmentation of the skin due to the loss of functional epidermal melanocytes. Epidermal melanocytes are responsible for the melanin synthesis which is driven by three key enzymes: tyrosinase (TYR), tyrosinase-related protein-1 (TYRP-1), and TYRP-2 in mammals [2]. The expression and activity of these three enzymes are regulated by the microphthalmia-associated transcription factor (MITF) [2]. The expression of the *MITF* gene can be modulated by many transcriptional factors in response to various environmental stimuli [3].

Aryl hydrocarbon receptor (AhR) is a ligand-dependent transcription factor that resides in the cytoplasm of various types of cells, including normal human epidermal melanocytes (NHEMs) [4]. Upon binding to ligands, AhR can translocate into the nucleus and regulate the expression of AhR-targeted genes such as cytochrome P450 1A1 (*CYP1A1*) [5]. In addition, AhR can also control the expression of other genes by interacting with various signaling pathways or regulating the half-life of other transcription factors [5]. It is well known that AhR is an important environmental sensor involved in a number of biological processes, including immune responses, endocrine regulation, and some chemical metabolism [5]. Our previous studies showed that AhR activation played roles in immune dysregulation and inflammatory response in CD4+ T cells and keratinocytes [6, 7]. Recently, AhR has been reported to modulate pigment synthesis via interacting with melanogenic signaling [4], and dysregulated AhR pathway might be involved in the development of vitiligo [8].

**Figure 1:**
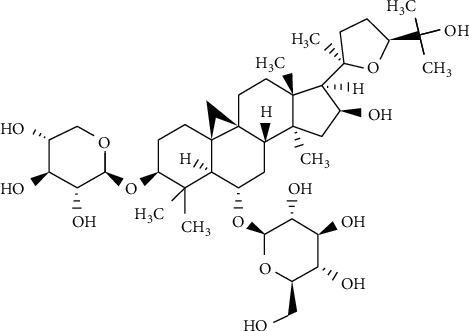
Chemical structure of AS-IV.

Many kinds of plant-derived products, for example, 8-methoxypsoralen (8-MOP), have been used for repigmentation treatment in vitiligo for thousands of years [9]. However, undesirable side effects of 8-MOP such as skin phototoxicity and risk of skin cancer limit its clinical applications [10, 11]. Therefore, it is desirable to find new products with better therapeutic effect and less side effects. *Astragalus membranaceus* is widely used in herbal medicine formulas such as Shouwushengheitang, Qubaixiaobantang [12], and Yiqiqubai [13] which have been successfully applied to treat vitiligo. Lin et al. [14] observed that aqueous extracts of the *Astragalus membranaceus* root were able to stimulate melanocyte proliferation. Astragaloside IV (AS-IV) is a main active component isolated from root of *Astragalus membranaceus* (Figure 1) [15]. Several studies have reported its pharmacological effects including antioxidation, anti-inflammation [15], antiscar [16], and anti-hair loss [17]. However, its effect on melanogenesis remains unknown.

Here, we aimed to evaluate the effect of AS-IV on melanogenesis in NHEMs and to explore the underlying mechanisms.

## 2. Materials and Methods

### 2.1. Cell Culture and Treatment

Primary human epidermal melanocytes (NHEMs) were isolated from human foreskin specimens obtained during circumcision surgery and cultured in Medium 254 (M254500) supplemented with HMGS-2 (S0163) and 1% antibiotic-antimycotic (Gibco, Grand Island, NY, USA). This study was approved by the ethics committee of Shanghai General Hospital (2017KY005) and conducted according to the principles of the Declaration of Helsinki. Cells were cultured in a humidified atmosphere with 5% CO_2_ at 37°C. Second- and fourth-passage NHEMs were used in all experiments. AS-IV (1044108), 6-formylindolo [3,2-b]carbazole (FICZ, SML1489), 8-MOP (M3501), AKT inhibitor VI (124013), FH535 (F5682), and CH223191 (C8124) were purchased from Sigma-Aldrich (St. Louis, MO, USA) and were dissolved in DMSO.

### 2.2. Cell Viability Assay

After treatment for 48 h, 10% of CCK-8 reagent (Yeasen, Shanghai, China) was added to each well and incubated for further 4 h at 37°C. The metabolic activation of CCK-8 was quantified by measuring the absorbance at 450 nm by using the spectrophotometer (Thermo Fisher Scientific Oy, Vantaa, Finland).

### 2.3. Melanin Measurement and Tyrosinase Assay

NHEMs were treated with AS-IV at concentrations of 0, 1, 10, and 100 *μ*M or 8-MOP at 100 *μ*M for 24 h. Cells were lysed and centrifuged to obtain supernatant for tyrosinase activity and precipitate for melanin content assays. Then, 30 *μ*g protein of the supernatant was mixed with 100 *μ*l 0.1% L-DOPA in PBS at pH 6.8 and incubated at 37°C for 30 min, and the tyrosinase activity was measured at 475 nm. The precipitate was dissolved in 100 *μ*l of 1 M NaOH with 10% DMSO for 1 h at 80°C to obtain melanin solution. The melanin content of melanin solution was measured at 405 nm.

### 2.4. Quantitative Polymerase Chain Reaction (qPCR)

Total RNA was extracted using RNAiso Plus (Takara, Kyoto, Japan) and was transcribed into cDNA using PrimeScript^TM^ RT Master Mix (Takara, Kyoto, Japan). The relative expression of various target genes was determined by q-PCR using TB Green® Premix Ex Taq™ II (Takara, Kyoto, Japan). Gene expression was normalized to that of *GAPDH*. Primer sequences used in this study are listed in Supplementary Table S1.

### 2.5. Western Blot Analysis

After treatment, NHEMs were lysed with RIPA Lysis Buffer or Nuclear and Cytoplasmic Protein Extraction Kit (Beyotime, Shanghai, China). About 20 *μ*g protein was separated by 10% SDS-PAGE and transferred to PVDF membranes (Merck Millipore, Billerica, MA, USA). After blocked in 5% bovine serum albumin (BSA, Yeasen, Shanghai, China) for 1 h, the membranes were incubated with primary antibodies overnight at 4°C and with secondary antibodies for 1 h. GAPDH and nup98 were used as the loading control. Bound antibodies were detected using the ECL western blotting detection system (Merck Millipore, Burlington, MA, USA). The information for primary antibodies used in the experiment is listed in Supplementary Table S2.

### 2.6. Transfection with AhR-Targeted Specific Small Interference RNA

AhR-siRNA (AM4611) and negative control siRNA were purchased from Ambion (Waltham, MA, USA). NHEMs were incubated with a mix of lipofectamine 3000 (Invitrogen, Carlsbad, CA, USA) and si-AhR (10 nM) or negative control siRNA for 24 h. Then, the NHEMs were treated with or without AS-IV for another 24 h.

### 2.7. Immunofluorescence

After treatment, NHEMs were washed once and fixed with 4% paraformaldehyde for 20 min, permeabilized with 0.3% Triton X-100 in PBS for 20 min, and blocked with 1% BSA for 30 min at room temperature. Samples were incubated with the anti-AhR (1 : 20) antibody at 4°C for 8 h and then incubated with the Alexa Fluor 488 secondary antibody (1 : 500, Invitrogen, Carlsbad, CA, USA) for 1 h at room temperature. Nuclei were stained with DAPI. Finally, images were captured using Leica TCS SP8 X Confocal (Leica, Mannheim, Germany).

### 2.8. Statistical Analysis

All data were presented as means ± SD and were analyzed using one-way ANOVA followed by Tukey's or Dunnett's multiple comparisons after the test. All analyses were performed using GraphPad Prism 7 software (San Diego, CA, USA). Differences were regarded as significant at *P* < 0.05.

## 3. Results

### 3.1. AS-IV Stimulated Melanin Synthesis in a Concentration-Dependent Manner in NHEMs

**Figure 2:**
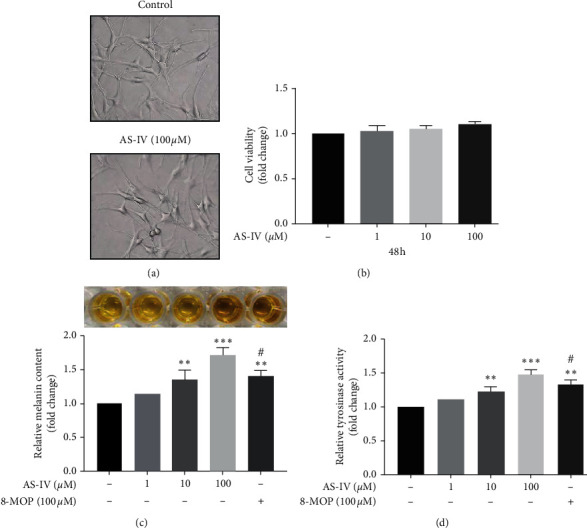
Effects of AS-IV on cell viability and melanogenesis in NHEMs. NHEMs were incubated with various concentrations (0, 1, 10, and 100 *μ*M) of AS-IV for 48 h for (a) morphology (magnification: 40x) and (b) cell viability; NHEMs were incubated with various concentrations (0, 1, 10, and 100 *μ*M) of AS-IV or 8-MOP (100 *μ*M) for 24 h for (c) melanin synthesis detection and (d) tyrosinase activity. Results were expressed as mean ± SD (*n* = 3) based on the representative experiment of at least three replicates. ^*∗∗*^*P* < 0.01 and ^*∗∗∗*^*P* < 0.001, versus untreated cells; ^#^*P* < 0.05, versus AS-IV-treated (100 *μ*M) cells according to one-way ANOVA followed by Tukey's multiple comparisons.

First of all, we detected the cytotoxicity of AS-IV on NHEMs. NHEMs were incubated with AS-IV at concentrations ranging from 0 to 100 *μ*M for 48 h, and then the viability of NHEMs was assessed using the CCK-8 cell viability assay. Our results showed that AS-IV had no cytotoxic effect on NHEMs and did not affect cell morphology (Figures 2(a) and 2(b)).

We then determined whether AS-IV could induce melanin synthesis in NHEMs. NHEMs were treated with various concentrations of AS-IV (0, 1, 10, and 100 *μ*M) for 24 h, and our results revealed that the melanin synthesis was increased by 15%, 35%, and 72% respectively. In addition, we also showed that tyrosinase activity was increased by 10%, 23%, and 48%, respectively. Furthermore, AS-IV at 100 *μ*M exhibited more effective in melanogenesis in NHEMs compared to 100 *μ*M 8-MOP (positive control, Figures 2(c) and 2(d)).

### 3.2. AS-IV Upregulated the Expression of Melanogenesis-Related Genes in a Concentration-Dependent Manner in NHEMs

**Figure 3:**
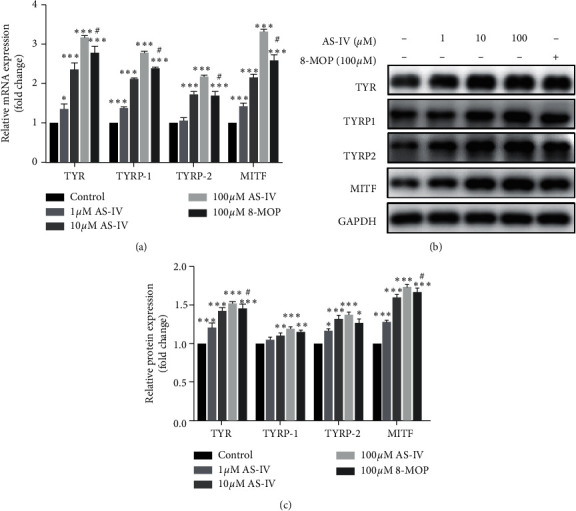
AS-IV upregulated the expression of melanogenesis-related genes in NHEMs. NHEMs were exposed to various concentrations (0, 1, 10, and 100 *μ*M) of AS-IV or 8-MOP (100 *μ*M) for 24 h for (a) qPCR, (b) western blot, and (c) quantitative analyses. Results were expressed as mean ± SD (*n* = 3) of one representative experiment of at least three replicates. ^*∗*^*P* < 0.01, ^*∗∗*^*P* < 0.01, and ^*∗∗∗*^*P* < 0.001, versus untreated cells; ^#^*P* < 0.05, versus AS-IV-treated (100 *μ*M) cells according to one-way ANOVA followed by Tukey's multiple comparisons.

To elucidate the mechanisms underlying AS-IV-mediated melanogenesis, we quantified the expression of TYR, TYRP-1, TYRP-2, and MITF. NHEMs were exposed to AS-IV (0, 1, 10, and 100 *μ*M) for 24 h, and the mRNA expression levels of TYR, TYRP-1, TYRP-2, and MITF were increased in a concentration-dependent manner. We also determined that the upregulation effect of AS-IV (100 *μ*M) on the expression of these genes was more robust than that caused by 100 *μ*M 8-MOP (Figure 3(a)). Likewise, western blot analysis demonstrated the protein expression levels of these genes were also upregulated in a concentration-dependent manner in NHEMs after 24 h treatment of AS-IV (Figures 3(b) and 3(c)).

### 3.3. AhR Signaling Was Essential for AS-IV-Induced Melanogenesis in NHEMs

**Figure 4:**
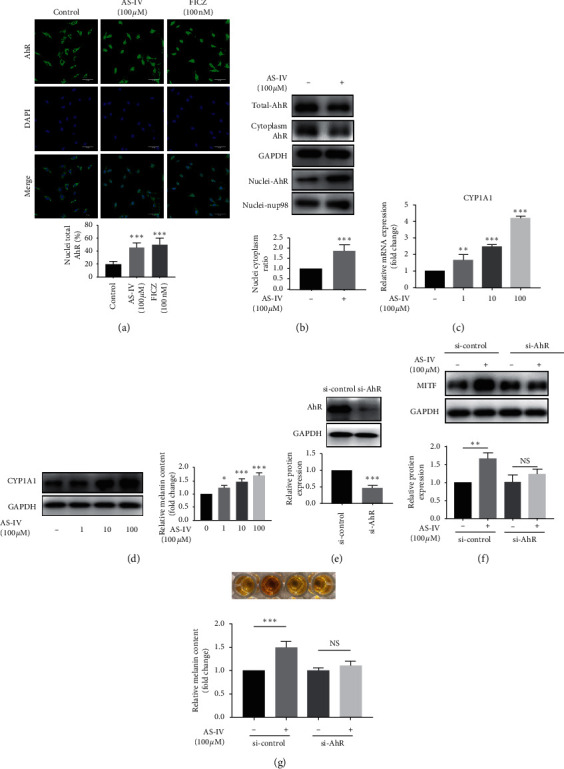
AhR signaling was essential for AS-IV-induced melanogenesis in NHEMs. NHEMs were incubated with AS-IV (100 *μ*M) or FICZ (100 nM) for 4 h. AhR nuclear translocation was detected by (a) confocal laser scanning microscopic analysis (scale bar = 50 *μ*m) and (b) western blot analysis and quantitative analysis. NHEMs were incubated with various concentrations (0, 1, 10, and 100 *μ*M) of AS-IV for 12 h or 24 h for (c) qPCR and (d) western blot and quantitative analyses. (e) The effect of si-AhR transfection. si-control and si-AhR-transfected NHEMs were treated with AS-IV at 100 *μ*M for 24 h for (f) western blot and quantitative analyses and (g) melanin content assay. Results were expressed as mean ± SD (*n* = 3), and images showed one representative experiment of at least three replicates. ^*∗∗*^*P* < 0.01 and ^*∗∗∗*^*P* < 0.001 according to one-way ANOVA followed by Dunnett's multiple comparisons.

Given that flavonoids are exogenous ligands of AhR [18], we reasoned that AS-IV might activate AhR in NHEMs. Therefore, we investigated whether AS-IV could lead to AhR nuclear translocation and increase the expression of AhR-targeted gene CYP1A1 in NHEMs. NHEMs were treated with AS-IV (100 *μ*M) or 6-formylindolo [3,2-b]carbazole (FICZ, 100 nM, positive control) for 4 h. Immunofluorescence staining showed that AS-IV clearly induced the translocation of the AhR protein into the nuclei, while AhR was mainly localized in the cytoplasm in an unstimulated condition (Figure 4(a)). Consistently, the protein expression of AhR was increased in the nuclei, while it decreased in the cytoplasm after AS-IV treatment for 4 h (Figure 4(b)). Moreover, the mRNA and protein expression of CYP1A1 were upregulated by AS-IV in a concentration-dependent manner in NHEMs after 12 h treatment (Figures 4(c) and 4(d)). These data suggested that AS-IV could activate AhR in NHEMs.

To validate the essential role of AhR in AS-IV-induced melanogenesis, we transfected NHEMs with si-AhR and then exposed the cells to AS-IV at 100 *μ*M for 24 h. As shown in Figure 4(e), the expression of AhR was successfully knocked down, and the increased MITF protein expression and melanin synthesis induced by AS-IV in the si-control condition were cancelled in the si-AhR condition (Figures 4(f) and 4(g)). These data indicated that AhR signaling was required for melanin synthesis induced by AS-IV in NHEMs.

### 3.4. AKT/GSK-3*β*/*β*-Catenin Pathway Was Involved in AS-IV-Induced Melanogenesis in NHEMs

**Figure 5:**
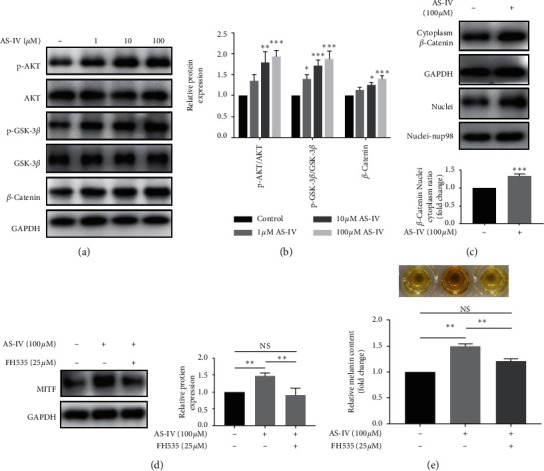
AKT/GSK-3*β*/*β*-catenin pathway was involved in AS-IV-induced melanogenesis in NHEMs. NHEMs were incubated with various concentrations (0, 1, 10, and 100 *μ*M) of AS-IV for 24 h, and the protein expression of p-AKT, AKT, p-GSK-3*β*, GSK-3*β*, and *β*-catenin was analyzed by (a) western blot and (b) quantitative analyses. NHEMs were incubated with or without AS-IV (100 *μ*M) for 24 h, and the protein expression of *β*-catenin in the cytoplasm and nucleus was analyzed by (c) western blot analysis and quantitative analysis. NHEMs were incubated with AS-IV (100 *μ*M) for 24 h with or without pretreatment with FH535 (25 *μ*M) for 3 h. Protein expression of MITF was analyzed by (d) western blot and quantitative analyses, and (e) melanin synthesis was detected. Results were expressed as mean ± SD (*n* = 3). ^*∗∗*^*P* < 0.01, ^*∗∗∗*^*P* < 0.001, and NS indicates nonsignificance according to one-way ANOVA followed by Dunnett's multiple comparisons.

Since AKT/GSK-3*β*/*β*-catenin signaling plays a critical role in the melanogenesis [19], we investigated the effect of AS-IV on this pathway. As shown in Figures 5(a) and 5(b), AS-IV significantly enhanced AKT and GSK-3*β* phosphorylation and increased the *β*-catenin protein expression level in a concentration-dependent manner in NHEMs after 24 h treatment. We also showed that the nucleus-to-cytoplasm ratio of the *β*-catenin protein was increased in NHEMs after AS-IV (100 *μ*M) exposure (Figure 5(c)). Furthermore, NHEMs were pretreated with *β*-catenin inhibitor FH535 (25 *μ*M) for 3 h and exposed to AS-IV (100 *μ*M) for 24 h. We demonstrated that the AS-IV-stimulated MITF expression upregulation and melanin synthesis were almost cancelled in the presence of the *β*-catenin inhibitor (Figures 5(d) and 5(e)). These results supported the idea that the AKT/GSK-3*β*/*β*-catenin pathway was involved in the AS-IV-induced melanogenesis in NHEMs.

### 3.5. AKT/GSK-3*β*/*β*-Catenin Pathway Was Controlled by AhR Activation in Response to AS-IV in NHEMs

**Figure 6:**
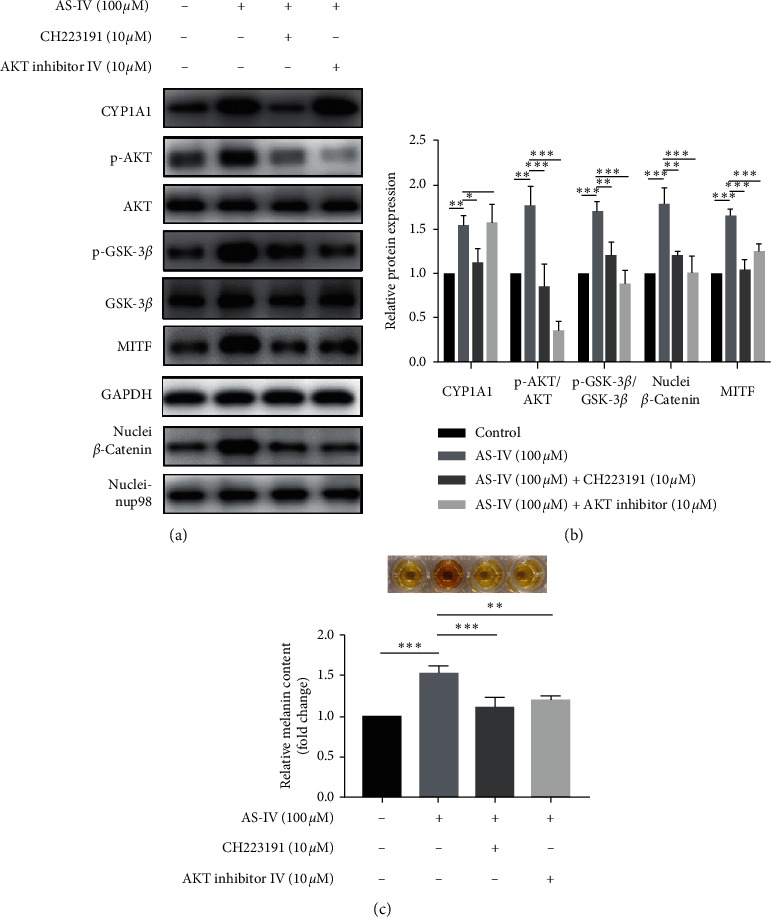
AKT/GSK-3*β*/*β*-catenin pathway was controlled by AhR activation in response to AS-IV in NHEMs. NHEMs were incubated with AS-IV (100 *μ*M) for 24 h with or without pretreatment with AKT inhibitor IV (10 *μ*M) or CH223191 (10 *μ*M) for 3 h. Protein expressions of CYP1A1, p-AKT, AKT, p-GSK-3*β*, GSK-3*β*, MITF, and nuclei *β*-catenin were analyzed using (a) western blot and (b) quantitative analyses; (c) melanin synthesis detection. Results were expressed as mean ± SD (*n* = 3). ^*∗∗*^*P* < 0.01 and ^*∗∗∗*^*P* < 0.001 according to one-way ANOVA followed by Dunnett's multiple comparisons.

To explore the crosstalk between AhR and AKT/GSK-3*β*/*β*-catenin signaling, NHEMs were pretreated with CH223191 (AhR inhibitor) or AKT inhibitor IV for 3 h and exposed to AS-IV (100 *μ*M) for 24 h. We observed that AS-IV-induced AKT/GSK-3*β*/*β*-catenin signaling activation and MITF protein expression upregulation were reversed under both CH223191- and AKT inhibitor IV-pretreatment conditions in NHEMs. In addition, we found that the protein expression of CYP1A1 was decreased in the presence of CH223191 but not affected by AKT inhibitor IV (Figures 6(a) and 6(b)). Similarly, the AS-IV-stimulated melanin synthesis was reduced in both CH223191- and AKT inhibitor IV-pretreated conditions (Figure 6(c)).

## 4. Discussion

In the recent past, AhR has been regarded as a novel regulatory factor of human melanin synthesis [20]. AhR^−/−^ mice showed lower tyrosinase activity [21], and mutations of the *AHR* gene were also associated with vitiligo susceptibility [22]. Consistently, lower transcription level of AhR was observed in the skin lesions of vitiligo patients [8]. These data imply that AhR may be a therapeutic target in depigmented skin diseases.

Many natural products can regulate melanogenesis through AhR activation [23]. AS-IV is a kind of natural flavonoids [24–26] which have been identified as exogenous ligands of AhR [27]. Currently, our study showed that AS-IV could stimulate the melanin synthesis via activating AhR in NHEMs.

**Figure 7:**
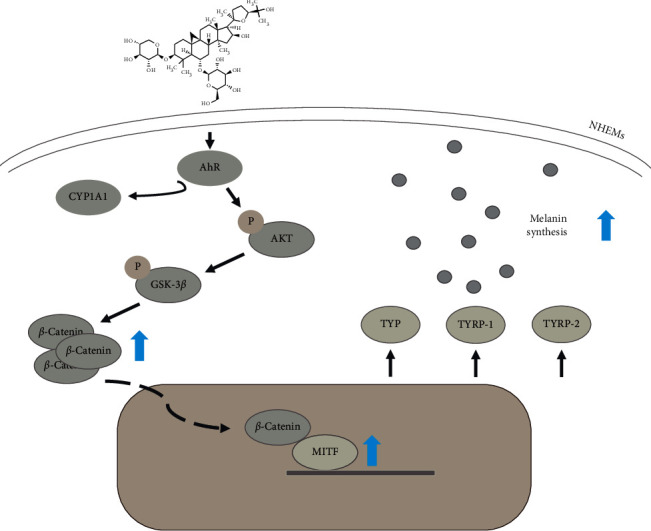
A proposed mechanism of AS-IV-induced melanogenesis. Astragaloside IV activates AhR, which then induces the phosphorylation of AKT and GSK-3*β* to promote *β*-catenin nuclear translocation. *β*-Catenin binds the promoter of the MITF gene to increase MITF transcription and subsequently upregulates TYR, TRP-1, and TRP-2 expressions and ultimately stimulates the melanin synthesis.

It has been reported that AS-IV could exert its pharmacological activities through the AKT/GSK-3*β*/*β*-catenin pathway [28, 29]. In the present study, increased phosphorylation levels of AKT and GSK-3*β* were identified in AS-IV-treated NHEMs. The phosphorylation of AKT increases GSK-3*β* phosphorylation, which further prevents *β*-catenin from degradation, increases its stability in the cytoplasm, and promotes its nuclear translocation [19, 30]. In line with this notion, increased nucleus-to-cytoplasm ratio of the *β*-catenin protein was detected in AS-IV-treated NHEMs. *β*-Catenin can further upregulate MITF transcription by binding to the MITF promoter together with LEF1 in nuclei, increases the expression of TYR, TYRP-1, and TYRP-2 [19, 31], and ultimately promotes the melanin synthesis (Figure 7).

Recently, several studies have focused on the crosstalk between AhR and other signal pathways [32–35]. It has been reported that TCDD, a high-affinity ligand of AhR, was able to increase the phosphorylation level of AKT and GSK-3*β* [36] and activate *β*-catenin [34]. Our study showed that AS-IV-induced AKT/GSK-3*β*/*β*-catenin pathway activation and melanin synthesis were abrogated with the presence of CH223191, whereas the AKT inhibitor IV could only inhibit melanin synthesis but had no effect on the upregulated CYP1A1 expression caused by AS-IV. These data suggested that the AKT/GSK-3*β*/*β*-catenin pathway might be the downstream signaling of AhR in response to AS-IV in NHEMs. The crosstalk between AhR and AKT/GSK-3*β*/*β*-catenin pathway is complicated and not fully understood. It has been reported that AhR could activate AKT in a ROS-dependent manner [37] or through binding Src kinase in the cell membrane [38]. In addition, the possibility that AhR triggers downstream effectors to promote *β*-catenin nuclear translocation or binds with *β*-catenin to move together into the nuclei cannot be excluded. Thus, the exact mechanism needs further investigation. It has been reported that *TYR*, *TYRP-1*, and *TYRP-2* genes might have multiple AhR-binding sites in their promoter regions [20]. In our study, we also showed that melanin synthesis induced by AS-IV was partially inhibited in the presence of AKT inhibitor IV raising the question whether AhR could induce melanogenesis through other pathways rather than AKT/GSK-3*β*/*β*-catenin signaling. This issue will be addressed in our future study.

There are some limitations in our study. NHEMs were used in our study as an *in vitro* model system, and further investigations on light- and dark-pigmented melanocytes are needed to provide comprehensive data on the effect of AS-IV. Moreover, although AS-IV stimulated melanin synthesis in our *in vitro* study, whether it induces normal skin hyperpigmentation *in vivo* needs more studies.

In conclusion, our study shows that AS-IV stimulates melanin synthesis in cultured NHEMs. This induction of melanogenesis by AS-IV is at least partially mediated by AhR-dependent AKT/GSK-3*β*/*β*-catenin signaling and the subsequent upregulated expression of MITF. Medications containing AS-IV might be beneficial in repigmentation for vitiligo and other acquired hypopigmentation diseases.

## Data Availability

The primer sequence and antibody data used to support the findings of this study are included within the supplementary information file.

## Supplementary Materials

Supplementary Materials Supplementary Table S1: primer sequences used for qPCR. Supplementary Table S2: antibodies used for western blot analysis.
