# Supplementary material for: Astragaloside IV Enhances Melanogenesis via the AhR-Dependent AKT/GSK-3β/β-Catenin Pathway in Normal Human Epidermal Melanocytes
Source: Evid Based Complement Alternat Med. 2020 Dec 15;2020:8838656. doi: 10.1155/2020/8838656 (PMC7755484; doi:10.1155/2020/8838656)
Supplement: Supplementary Materials — Supplementary Table S1: primer sequences used for qPCR. Supplementary Table S2: antibodies used for western blot analysis. [file 8838656.f1.docx]

**Supplementary Table S1. Primer sequences used for qPCR.**

| Primer name | Sequence (5'-3') |
| --- | --- |
| MITF FW | CTCACAGCGTGTATTTTTCCCA |
| MITF RV | ACTTTCGGATATAGTCCACGGAT |
| TYR FW | TCAGCACCCCACAAATCCTAA |
| TYR RV | AATCGGCTACAGACAATCTGC |
| TYRP-1 FW | TCTCTGGGCTGTATCTTCTTCC |
| TYRP-1 RV | GTCTGGGCAACACATACCACT |
| TYRP-2 FW | AACTGCGAGCGGAAGAAACC |
| TYRP-2 RV | CGTAGTCGGGGTGTACTCTCT |
| CYP1A1 FW | ACATGCTGACCCTGGGAAAG |
| CYP1A1 RV | GGTGTGGAGCCAATTCGGAT |
| GAPDH FW | CATGTACGTTGCTATCCAGGC |
| GAPDH RV | CTCCTTAATGTCACGCACGAT |

**Supplementary Table S2. Antibodies used for Western blot analysis.**

| Antibodies | Product code |
| --- | --- |
| Anti-AhR | ab190797 |
| Anti-CYP1A1 | ab3568 |
| Anti-TYRP-1 | ab178676 |
| Anti-TYRP-2 | ab74073 |
| Anti-MITF | ab12039 |
| Anti-AKT | #5106 |
| Anti-p-AKT (Thr308) | #5373 |
| Anti-GSK-3β | #9832 |
| Anti-p-GSK-3 (Ser9) | #9323 |
| Anti-β-catenin | #8480 |
| Anti-nup98 | #2598 |
| Anti-TYR | sc7833 |
| Anti-GAPDH | sc47724 |
